# Motivation in the age of genomics: why genetic findings of disease susceptibility might not motivate behavior change

**DOI:** 10.1186/2195-7819-9-8

**Published:** 2013-08-19

**Authors:** Tinsley HG Webster, Sarah J Beal, Kyle B Brothers

**Affiliations:** Center for Biomedical Ethics and Society, Vanderbilt University, Nashville, TN USA; Division of Adolescent Medicine, Cincinnati Children’s Hospital Medical Center, Cincinnati, OH USA; Department of Pediatrics, University of Louisville, 231 East Chestnut Street, N-97, Louisville, KY 40202 USA; Institute for Bioethics, Health Policy, and Law, University of Louisville, Louisville, KY USA

**Keywords:** Genomics, Incidental findings, Actionability, Human motivation

## Abstract

There is a growing consensus that results generated through multiplex genetic tests, even those produced as a part of research, should be reported to providers and patients when they are considered “actionable,” that is, when they could be used to inform some potentially beneficial clinical action. However, there remains controversy over the precise criterion that should be used in identifying when a result meets this standard. In this paper, we seek to refine the concept of “actionability” by exploring one proposed use for genetic test results. We argue that genetic test results indicating that a patient is at risk for developing a chronic health condition should not be considered actionable if the only potential value of that result is to motivate patients to make changes in their health behaviors. Since the empirical research currently available on this question is equivocal, we explore relevant psychological theories of human motivation to demonstrate that current theory does not support the assumption that information about genetic risk will be motivating to most patients in their attempts to make changes in health behaviors.

## Introduction

Scientists, clinicians, and policy-makers have aspired for a decade to improve healthcare by using multiplex genetic technologies to personalize and stratify the prevention, diagnosis, and treatment of illnesses (Simmons et al. 2012). Although several institutions have begun to incorporate principles of Personalized Medicine into clinical care in specific programs (Pulley et al. 2012), the possibilities for improving care through Personalized Medicine across the population remain nascent. For now, diverse clinical and translational research efforts are underway to explore how providers in a range of settings might be able to improve care through the application of Personalized Medicine.

Given the transitional state of Personalized Medicine, it is perhaps not too surprising that institutions, providers, and even researchers are frequently facing dilemmas about when particular uses of advanced laboratory modalities are “ready for prime time.” (Shurin & Nabel 2008; Berg et al. 2011; Li-Wan-Po et al. 2010) In fact, the intense debate on the return of genomic research results can be interpreted largely as a debate about the readiness of genetic laboratory results for clinical use, since much of that debate has centered on the issue of when a result should be considered “actionable.” (Wolf et al. 2012; Clayton 2012; Ravitsky & Wilfond 2006)

Importantly, both research studies and clinical programs are already producing large volumes of genetic results. Investigators and clinicians are actively facing decisions about the proper use for the genetic findings they are producing. For this reason, efforts to clarify the criteria for actionability are somewhat urgent. In this paper, we seek to advance those efforts in one specific area. We argue that when a genetic test result produced through clinical or research testing indicates that a patient is at risk for developing a chronic health condition, that finding should not be considered actionable if its only potential value is to motivate patients to make changes in their health behaviors.

Of note, we are not the first to seek clarification of the criteria for actionable genetic results. A working group convened in 2009 by the National Heart, Lung and Blood Institute to provide guidance on when genetic research results should be returned defined an actionable result as one that “has the potential to lead to an improved health outcome; there must be established therapeutic or preventive interventions available or other available actions that may change the course of disease.” (Fabsitz et al. 2010) Many have joined this group in proposing that actionable research results carry adequate utility to justify reporting them to patients and providers (Keller et al. 2010; Fullerton et al. 2012; Wolf et al. 2012). The actionability standard has more recently been appropriated in discussions on the use of genetic findings incidentally produced through *clinical* genotyping (Fullerton et al. 2011).

Despite the growing consensus that genetic results generated through either clinical or research tests should be reported to providers and patients when they are considered actionable, there remains controversy over the precise criterion that should be used in identifying when a result meets this standard. Presently, actionability is usually identified through expert deliberation on a case-by-case basis (Fullerton et al. 2012). Efforts are underway, however, to generate more precise criteria and apply those criteria in a structured way (Evaluation of Genomic Applications in Practice and Prevention EGAPP 2012; HuGE Navigator - Genopedia - Search 2012).

One conflict that these efforts will need to negotiate is the apparent contrast between the actionability standard and the standards of evidence-based medicine. Evidence-based medicine aims to inform clinical decisions using outcomes-based empirical research. “Evidence-based medicine de-emphasizes intuition, unsystematic clinical experience, and pathophysiologic rationale as sufficient grounds for clinical decision making.” (Guyatt et al. 1992) Proponents of actionability, on the other hand, recognize that empiric evidence is not always available. Clinical decisions, especially those related to the use of emerging technologies, must sometimes be based on a careful consideration of available pathophysiological understanding and clinical experience (Burke et al. 2002). Most proponents of the actionability standard seem to acknowledge the importance of systematic observations for guiding routine clinical practice, but recognize at the same time that clinicians must make the most of the information and experience that is available in the present.

### Genetic risk and patient motivation: evidence base

There has been a great deal of hope that genetic testing could be used to improve the treatment and prevention of such chronic health conditions as coronary artery disease, type 2 diabetes, obesity, and cancer. The hope that principles of Personalized Medicine may be effective in the prevention and treatment of these conditions, the most common and most costly diseases in the developed world, is based partly on the recognition that most of them result from the interaction of genetic and behavioral factors. Even though the genetic contribution to disease is not regarded as modifiable, health behaviors seem to offer an excellent opportunity for modifying risk. Scientists and healthcare providers have expressed interest in motivating at-risk patients to make health behavior changes by providing them with information about their genetic risk (Schaumberg et al. 2007; Gramling et al. 2003; Collins et al. 2003; Hernandez 2005; Christensen & Green 2013). Although such behavior changes could not reverse genetic risk, they could potentially mitigate the overall risk of disease.

Expectations that genetic risk information could be motivating to patients have already been tested across a range of health behaviors and disease risks. Intention to change has been shown to be a strong predictor of actually making a behavior change, and several studies have indeed demonstrated that some individuals respond to genetic risk information with increased intention to make behavior changes (Marteau & Lerman 2001). Investigators have also examined whether information about genetic risk for developing lung cancer will cause smokers to demonstrate cognitions and emotions that favor smoking cessation (Lerman et al. 1997; Wright et al. 2006). Others have studied whether knowledge of genetic variants that increase risk for obesity, either in real patients or in case vignettes, will improve confidence or intentions to get more exercise and eat healthier foods (Frosch et al. 2005; Harvey-Berino et al. 2001).

The precise empirical question in the context of genetic test results, however, is whether being informed of a high-risk genetic variant will actually increase success at making behavior changes compared with those who are not provided genetic risk information. Only four studies have collected data that can be used to answer this question directly (Table 1) (Chao et al. 2008; Ito et al. 2006; McBride et al. 2002; Sanderson & Michie 2007), one of which examined more than one health behavior (Chao et al. 2008). Of the three studies that examined smoking cessation, two demonstrated that those informed of increased genetic risk were more likely to quit smoking compared with those provided with no genetic risk information (Ito et al. 2006; McBride et al. 2002). However, a pooled analysis of all three of these studies indicated no effect from reporting positive risk information compared with control groups (pooled OR 1.51, 95% CI 0.88 – 2.61) (Marteau et al. 2010). The only study that did not look at smoking cessation showed no significant increase in health behaviors associated with Alzheimer’s Disease among those informed they were at risk for this condition (Chao et al. 2008). Importantly, the four original studies (and pooled analyses subsequent studies relied on) were limited in scope, with samples that were underpowered and biased toward higher motivation to receive genetic testing. As a result, few empirically-supported conclusions can be made about motivation based on genetic information.

**Table 1:** Studies empirically comparing health behavior changes among those informed of a high-risk genetic variant compared with those provided no genetic risk information*

Publication	Health behavior	Follow-Up period	N**	OR (95% CI)**	Overall finding
McBride 2002	Smoking Cessation	Sustained at 6 & 12 months	104 vs. 185	2.79 (1.15 – 6.78)	Positive
Ito *et al*. 2006	Smoking Cessation	9 months	167 vs. 356	1.78 (1.12 – 2.83)	Positive
Sanderson 2008	Smoking Cessation	2 months	20 vs. 18	1.40 (0.35 – 5.57)	Negative
Chao 2008	Diet Changes	12 months	50 vs. 51	3.33 (0.85 – 13.02)	Negative
Chao 2008	Physical Activity	12 months	50 vs. 51	1.78 (0.31 – 10.25)	Negative
Chao 2008	Medication/Vitamin Use	12 months	50 vs. 51	1.72 (0.72 – 4.13)	Negative

*Some values not provided in original publication, but reported by authors to a workgroup conducting a Cochrane Collaboration review. These findings were reported in Marteau 2010.

** Elevated risk group vs. no genetic information (control) group.

When empirical data is incomplete, groups who conduct evidence-based reviews generally include a lowest level of evidence quality that depends on expert opinion (U.S. Preventive Services Task Force Procedure Manual 2011) or “mechanism-based reasoning.” (Howick et al. 2011) In this case, the relevant mechanism we need to reason about is a psychological rather than physiological one. We need to reason from the best available theory on human motivation in order to determine whether being informed of genetic risk for developing a disease is likely to be motivating for patients.

In this paper, we will argue that leading theories and models of human motivation do not support the use of genetic risk information to motivate changes in health behaviors. In order to show why this is so, we will briefly review and critically evaluate three such theories that have been developed in the fields of psychology and education. Although these theories represent human motivation and behavior using different overall schema, they do provide some consensus on specific points. More importantly, the robust theoretical and empirical grounding of these theories serve to refute reductive views of human motivation and behavior. Ultimately, we will show that these three theories, taken together, raise significant doubt about the plausibility of the expectation that genetic risk information is clinically “actionable” on the basis of expectations that it may provide motivation to patients.

### Theories of human motivation: which one is *right*?

Scholars in the fields of psychology and education have developed a number of models to illuminate how and why humans are motivated to act. Many of these are intended to predict behavior in very circumscribed situations, while others are intended to describe motivation in a wide range of settings. In addition to differences in scope, these theories also differ in the psychological constructs that are included as relevant within their schema. Contrasting theories sometimes focus on seemingly different constructs, but more often these constructs are fundamentally similar and their disparity lies primarily in nuance of description or relationship to the overall schema.

Given the prominent role in science for using experimentation to select between competing theories about biological or chemical processes, it is tempting to view these theories as competitors. In one sense, they are. As one model or theory gains popularity through successful outcomes in the lab, it also gains credibility as a plausible explanation for human behavior. Findings that only partially support the theory being tested lead to revisions in those theories; some of the most robust theories have evolved substantially through this refinement process.

However, these theories or models of human behavior are not always in strict competition. As mentioned before, they frequently overlap, or describe different aspects of the same construct, or even describe the same construct in different language (Ajzen 1998; Armitage & Conner 2000; Noar & Zimmerman 2005; Weinstein 1993; Weinstein & Rothman 2005). Given the problem of potential construct overlap, some experts have called for studies that not only pit multiple theories against each other, but also compare individual theories and models with a single theory that incorporates the most promising and robustly supported elements from among the group (Noar & Zimmerman 2005; Weinstein 1993; Weinstein & Rothman 2005; Gerend & Shepherd 2012).

Extant comparative research is scarce and imperfect. By one report, there have been only 11 longitudinal studies in the last two decades comparing motivation theories in the arena of health behavior research (Brewer & Gilkey 2012). Another review examined 19 comparative studies and suggested a number of directions for future research, including meta-analysis and the investigation of multiple behaviors across age groups (Noar & Zimmerman 2005). Others have noted that many comparative studies inappropriately rely on tests of explained variance and suggest instead that researchers should adopt consistent and inclusive criteria to define the superiority of a theory (Weinstein & Rothman 2005).

Notwithstanding these significant areas of controversy in the field, we have selected just three theories for closer inspection. Although each of these theories is still evolving, each has been used widely in the study of health behavior and each is supported by a set of empirical literature. The three models of motivation we will explore below share many similarities: they were all developed within the social-cognitive tradition in psychology; they are all considered expectancy-value models of motivation; and they each underscore the importance of individual perception—perception of control, self-efficacy, or causal explanations—in the generation and maintenance of human motivation.

We selected these models because they have figured prominently in the discourse on health behavior and motivation over the last thirty years. We view these as distinct but related theories, each providing its own contribution to our understanding of human motivation. In our discussion, we will emphasize the manifold complexities of abstract human behavior and the study of it, while at the same time highlighting the way each theory or model can help geneticists and healthcare providers anticipate the efficacy of using genetic risk feedback to promote behavioral change in patients.

### The health belief model

The Health Belief Model (HBM) (Rosenstock 1974; Strecher & Rosenstock 1997) was first developed to explain why patients adopt or fail to adopt preventative health measures. It has since come into wide use in genetic counseling and other fields to guide the development of approaches to motivate patients.

The central tenet of this model is that adults are motivated to protect themselves against risk. The model was originally formulated to include four dimensions that together were thought to comprise motivation for health behavior:

- *Perceived susceptibility* is an individual’s evaluation of the likelihood of a negative outcome occurring (Strecher & Rosenstock 1997). For example, a smoker is likely to develop an opinion about the likelihood that he or she will develop lung cancer. This evaluation may be based on a wide range of factors, including messages in the media and experience with family members.
- *Perceived severity* refers to a patient’s understanding of the consequences of an outcome, including the consequences of neglecting treatment or prevention (Strecher & Rosenstock 1997). For the smoker, it may entail imagining how much he may suffer if he develops lung cancer, how much his personal relationships or career may be affected, and how much it will cost to undergo treatment.
- *Perceived benefits* are the patient’s notions of the benefit or efficacy of treatment or behavior change in combating risk (Strecher & Rosenstock 1997). For example, should the smoker determine that he is at risk for lung cancer, he must evaluate whether smoking cessation is likely to mitigate his chances of developing illness.
- *Perceived barriers* are the “costs” of a treatment or behavior change weighed against the benefits (Strecher & Rosenstock 1997). These are the personal, environmental, financial, or cultural obstacles that prevent the patient from adopting behavior change or choosing treatment. For the smoker, one barrier might be the salient, short-term pleasure of smoking weighed against the less determinate future of disease.

Notice the repetition of the word *perceived*. It is an individual’s subjective analysis of her own person, vulnerability, and future self that influences her decision to act or not to act. Because of the importance of perception in this model, changes in relevant health behaviors (e.g. smoking) in response to information about elevated risk are not assured. It seems reasonable to expect that a patient’s perception of susceptibility would increase upon hearing that she is at increased genetic risk to develop a disease, and that this would motivate healthy changes in behavior. If this is true, then we would also expect that a patient would be *demotivated* to change behaviors if she learns that she *is not* at risk. This is because her perception of susceptibility would be decreased rather than increased. A closer examination of the HBM reveals that both expectations are problematic.

### Why risk feedback might not lead to motivation

According to the HBM, the key to motivation lies in the patient’s own perceptions of risk in conjunction with his estimation of the likelihood that behavior change will result in the mitigation of the risk (Rosenstock 1974; Strecher & Rosenstock 1997). For this reason, telling a patient that she is at a moderately increased risk for developing a disease, such as that conferred by a genetic risk variant, is unlikely to lead to behavior change unless (1) this information substantially increases the patient’s perception of risk and (2) the patient believes that behavior change will effectively mitigate this risk. Perceived barriers to behavior change may still override the patient’s assessment of risk or benefits of change. For example, a person may feel that starting an exercise regimen would decrease her likelihood of obesity, but she may be prevented from pursuing this behavior because she also perceives that exercise is inconvenient or joining a gym is too costly. Therefore, it cannot be assumed that genetic feedback indicating high risk for disease would, for this patient, overcome the barriers that are already in place against behavior change.

Indeed, a recent meta-analysis of studies that tested the original version of the HBM revealed that perceived barriers and benefits were the most significant predictors of behavior change, while susceptibility and severity contributed little or no effect (Carpenter 2010). Other analyses of the HBM stress that the four main dimensions of the HBM do not, individually, carry much predictive power; rather it is their combination that is most revealing (Conner 1998). In summary, then, we cannot infer from current evidence that patients’ perceived susceptibility or severity of illness contributes much to their motivation to change health behavior, nor that these perceptions can be altered by the provision of new information.

### Criticism of the HBM

A number of methodological concerns have been raised about studies examining the validity of the HBM. These include inconsistent operationalization of terms across studies, lack of combinatorial rules showing the relationship between constructs, and failure to use validated measures to capture the various dimensions of the HBM (Weinstein 1993; Carpenter 2010; Janz & Becker 1984). In addition, longitudinal and prospective research on the HBM remains scarce (Carpenter 2010). Finally, even proponents of the HBM have acknowledged that the four dimensions proposed in the original model paint an incomplete picture of motivation, and that other dimensions, such as perceived self-efficacy to change behavior, should be included (Rosenstock et al. 1988).

Despite this complex picture, we perceive a trend in the discourse on the clinical utility of genetic information to overemphasize susceptibility in the application of the HBM. While we expect that the HBM will continue to contribute to the development of a wide range of successful interventions in the Era of Personalized Medicine, we fear that unjustified focus on perceived susceptibility is driving overly optimistic estimates of the relevance and importance of genetic risk information in specific clinical settings. Current empirical evidence based on the HBM does not support the otherwise intuitive notions that perceived susceptibility can be increased with genetic risk information and that this change would be likely to lead to a change in health behaviors.

### Theory of planned behavior

The Theory of Planned Behavior (TPB) suggests that the central component of motivation is the *intention* to act, which is positively correlated with actually performing a specific action (Ajzen 1991). In other words, the greater a person’s intention, the more likely he is to act. Intentions are influenced by at least three constructs: attitudes, subjective norms, and perceived behavioral control (Ajzen 1991), each of which may be composed of two subcomponents (Rhodes & Courneya 2003; Hagger & Chatzisarantis 2005; Ajzen 2002). There is disagreement about the relationship between these subcomponents and among the three primary constructs. Some favor a hierarchical model (Hagger & Chatzisarantis 2005; Ajzen 2002) and others argue for a multidimensional model (Rhodes & Blanchard 2006; Trafimow et al. 2002). The importance of additional constructs that may contribute also remains controversial.

- *Attitudes* emerge from behavioral beliefs, or beliefs about behaviors, especially their associated outcomes and costs. These beliefs often require that we draw on information that is linked to particular feelings and valuations, and because of this, we develop attitudes about people, events, and behaviors. These attitudes inform our intentions to act, and, according to TPB, the strength of our attitudes determines the likelihood that we will act on our intentions (Ajzen 1991). Attitudes can either be instrumental, i.e. the behavior is harmful/beneficial; or affective, i.e. the behavior is enjoyable/unenjoyable (Rhodes & Courneya 2003; Hagger & Chatzisarantis 2005).
- *Subjective norms* stem from underlying normative beliefs, which may be thought of as an individual’s perceptions of the rules of behavior imposed by friends, family, or society in general. Our willingness to comply with these social expectations or pressures determines their influence on our intentions (Ajzen 1991). Subjective norms include injunctive and descriptive norms (Rhodes & Courneya 2003; Hagger & Chatzisarantis 2005). For example, a patient may believe that her husband would like her to stop smoking (injunctive norm); or she may observe that all the members of her social network are nonsmokers (descriptive norm). If she is willing to comply with the social pressure applied by these norms, TPB predicts that she is more likely to change her smoking behavior.
- *Perceived Behavioral Control (PBC)* is derived from control beliefs, or the beliefs about the controllability of a behavior. An assessment of controllability requires an individual to make judgments about the difficulty of performing the behavior, drawing on past experience or the reported experience of others. While PBC is arguably the most important component of behavioral intentions (Armitage & Conner 2001), there has been some debate about how exactly PBC should be operationalized. Azjen (Ajzen 2002) suggested that PBC should be thought of as an individual’s perception that he can control a specific behavior, and that it is related to Bandura’s conception of self-efficacy (Bandura 1977). Others have argued that studies that measure PCB are actually measuring two distinct constructs: self-efficacy, i.e. an individual’s belief in his ability to perform certain behaviors, and perceived control, i.e. the individual’s belief a behavior is, in general, controllable by an actor (Armitage & Conner 1999; Terry & O'Leary 1995). There is evidence that self-efficacy in particular contributes to intentions and behavior more significantly than controllability, attitudes, and subjective norms (Ajzen 2002; Trafimow et al. 2002; Armitage & Conner 2001).

In summary, it is the individual’s beliefs about her ability, the effort required, the characteristics and costs of behavior, the expected outcomes of her behavior, and her perception of the controllability of behaviors that are central to her intentions, motivation, and action.

### Genetic risk feedback and perceived control

The TPB does not support the expectation that genetic risk feedback, *per se*, will motivate or demotivate patients. For example, imagine a man who is told that he carries a genetic variant that places him at greater risk for developing obesity, which is often considered a disease that can be controlled by changes in behavior. If we apply the TPB to this scenario, we see that knowledge of genetic risk only motivates the obese patient to exercise if it significantly alters his attitudes and normative assessments of, or perceived control over, exercise activities. By itself, the information is neither motivating nor demotivating: such power is conferred by its integration into a pre-existing network of attitudes, values, and assessments of behavioral control. Does he feel pressured by friends or family to exercise? Does he feel an increase in his activity would effect change in his weight? If so, does *he* feel capable of controlling his exercise habits? After all, a patient may agree that people in general can control their workouts, but he may not feel that *he*, specifically, has any control over this. These issues are as much present for the patient who is given genetic feedback as they are for the patient who is given general information about the risk of unhealthy behaviors.

Framing genetic risk feedback in the schema of this motivation model, we can see that the main issue is what effect knowledge of increased risk will have on perceived behavioral control. In particular, it is possible that patients will interpret a very elevated genetic risk deterministically, and that this will lead to a *loss* of perceived control and an increase in fatalism. In one study, women were more likely to report low perceived behavioral control after they were told they had increased genetic risk for obesity in a hypothetical testing scenario. For women who had family histories of obesity and high BMIs, in addition to low PBC, feedback of increased genetic risk resulted in low intentions to eat a healthy diet. The authors concluded that these results could indicate that subjects had a deterministic view of genetics. In the case of women who were already overweight or had family histories of obesity, such views may have caused feelings of fatalism (Frosch et al. 2005). It should be noted, however, that this study could not show the direction of this effect: genetic determinism might precede low PBC, but it is also possible that low PBC with respect to eating habits contributed to the view that genetics are deterministic.

Analyses of TPB show that PBC accounts for a significant amount of the variance in intention and behavior change (Armitage & Conner 2001). This observation, combined with the current inability of clinicians to predict who will respond deterministically or fatalistically to risk feedback, support a cautious approach in considering whether obese patients, for example, should intentionally be provided information about their genetic predisposition to obesity. If a patient already has insufficient attitudes or control beliefs about exercise to effect behavior change, then there is no reason to suppose that knowledge of genetic risk will meaningfully shift her current motivation. Quite the opposite seems to be the case. If a patient told she has a genetic predisposition to obesity responds by suffering a loss of perceived control over weight loss, TPB suggests that a decrease in intentions to change behavior is likely to result (Ajzen 1991). In this case, not only would no benefit have been gained, but also the patient could actually be harmed. Given the framing provided by TPB, then, there is no compelling reason to deliver risk feedback for the purposes of motivating behavior change.

### Attribution theory

Unlike the TPB or the HBM, which aim to predict future behavior, attribution theories are concerned with the causes people attribute to past events, especially negative or unexpected events (Weiner 1985). Attribution theories suggest that humans organize their experience of the world, in part, by generating an answer to the question, “Why did this happen to me?” To answer this question, an individual will ascribe causes to events or outcomes, and the specific qualities of these causes determine both the individual’s present affect and future behavior (Weiner 1985). Attribution theories have been influential in education, marketing, and coping research over the last 30 years, and have played an important role in the development of the learned helplessness theory of depression (Abramson et al. 1978; Alloy et al. 1984; Seligman et al. 1979).

### Explaining failure

According to one prominent articulation of an attribution theory of motivation (Weiner 1985), the process of assigning causal attributions is a multi-stage process that begins with an event. First, the individual experiences emotions related to the event or outcome. If the event is unexpected or negative, she might feel sad or discouraged. Second, she will search for the causes to explain why the event occurred. This search will depend on her perceptions of *causal antecedents*, which include an individual’s personal history and the social norms that influence her (Weiner 1985; Weiner 2010).

Causes are composed of two main features, *causal ascription* and *causal dimension.* Causal ascriptions are the answers to the question, “Why did this happen to me.” Ability, luck, and effort, are three examples of causal ascriptions (Weiner 1985; Weiner 2010). An individual’s understanding of the causal ascriptions she favors is characterized by four causal dimensions:

- *Causal locus* refers to an individual’s perception of whether the cause comes from an internal or external source.
- *Causal stability* relates to one’s perception of whether a cause is stable or unstable across time.
- *Causal controllability* is determined by one’s perception of how much control he or she has over a cause (Weiner 1985).
- The learned helplessness theory of depression adds a fourth causal dimension, *causal globality.* (Abramson et al. 1978) Some persons tend to globalize causal dimensions across situations, while others understand them to be specific to a particular situation only (Abramson et al. 1978).

It is important to note that while theorists agree that we generally choose from the same set number of causal ascriptions, we don’t all locate these in the same dimensional space. For example, one patient might attribute her failed attempt to lose weight to low ability, which she perceives to be an internal, stable cause that she cannot control. Another patient might also attribute her failure to lose weight to low ability, but instead believe that ability level is unstable and controllable. These individual differences are key, because arguably the most important phase of the causal search is the cause-dependent affect, or the emotional consequences of an individual’s determination about where a cause lies in the causal dimensions.

Research has shown that stable, internal, and uncontrollable causal attributions are associated with feelings of depression, shame, helplessness, and hopelessness (Weiner 1985; Abramson et al. 1978; Seligman et al. 1979). Moreover, these negative emotions are thought to be the source of demotivation. In other words, the hopeless patient has little expectancy that further effort will be successful; therefore she fails to persist in effortful behavior. In addition, persons will vary in their behavioral and emotional responses to dimensional characteristics; while some may be spurred on by guilt caused by attributing failure to a controllable cause, others may feel paralyzed (Weiner 1985).

Again, it is the patient’s perception of a cause’s characteristics, or causal dimensions, that are integral to his behavior change, not the researcher’s or physician’s perception. Regardless of whether empirical research supports the idea that diet and exercise are controllable, such a designation is only meaningful if it comes from the patient. As we have noted, the same cause might take on different meanings for different individuals. What is meaningful in an analysis of behavior based on attribution theory is the correlation among the dimensional characteristics of causes, e.g. controllability, globality, externality, and behavior. Without this information, it is impossible to pinpoint why a patient who believes healthy diet prevents heart attack nevertheless neglects to change his diet; knowing that the patient does not believe that *he* can control *his* diet is crucial to our understanding of his behavior.

### Attribution theory and genetic test results

It is clear that genetic information can be received in a number of ways. Upon receiving genetic information related to disease risk, a patient may perceive the causal attributions related to this information to be uncontrollable, stable, and internal. Such dimensional characteristics could lead to feelings of low self-esteem and helplessness, and this combination could demotivate the patient. On the other hand, a different patient might process the same information quite differently. He might consider his disease risk controllable, for example if he focuses on the role environment plays in gene expression. Again, even though medical providers and genomic researchers may enjoy a relatively strong consensus on the stability and controllability of genetic variants and genetic risk, we cannot assume that patients share the same scientific understanding.

Moreover, learned helplessness research has focused on the role attributional style plays in the onset of depression or helplessness. (Seligman et al. 1979; Anderson et al. 1994; Romens et al. 2011) Individuals who consistently attribute failures to causes that are uncontrollable, internal, and global are more likely to become depressed, but they are also more likely to attend to negative stimuli in their environment. These individuals are attuned to any message, external or internal, that reinforces their attributional style. They are biased toward negative or threatening information in their environment, and they tend to see ambiguous information in a negative light (Romens et al. 2011). Given that genetic risk feedback hardly communicates certainty, it is possible that a person with negative attributional style would be more likely to interpret this information in the least adaptive way.

### Considering life stage and future orientation

There are at least two additional aspects of human motivation and predictions of behavior that are generally not taken into account by these or other expectancy-value models, but which are important to our analysis of the utility of genetic information: life stage and future orientation. These concepts together help illuminate why individuals are likely to vary when it comes to being motivated by genetic information.

The concept of future orientation has been examined for decades, and encapsulates the notion that individuals vary in the extent to which they consider a future state when engaging in present behaviors (Massey et al. 2008). Findings from this literature would suggest that individuals will be more motivated by proximal (i.e., close to the present) outcomes rather than distal (i.e., far from the present) outcomes, with some variation across individuals (Lens & Moreas 1994). As such, an individual will generally be more motivated to engage in behaviors with immediate benefits than benefits that they cannot see or experience for a lengthy period of time. Notably, most genetic findings fit into this latter category. In order for genetic information to motivate present behaviors, an individual would likely need to develop an elaborate conceptual sense of a self in the future who has the disorder or diagnosis the genetic finding indicates they are at risk for, and then be motivated to avoid that future state, or feared self (Markus 1986). Developing such an orientation to the future is not a process that is likely to occur automatically when genetic findings are given. Rather, most patients are likely to require significant assistance in developing such a future orientation.

Importantly, the process of developing a future orientation, and motivation to avoid future risk over more proximal gains, differs dramatically across development from adolescence to adulthood. There is a large body of evidence indicating that adolescents are especially unlikely to be motivated by avoiding negative outcomes, but are instead more oriented toward taking risks to receive an immediate or proximal reward (Steinberg 2006; Steinberg 2008; Steinberg 2005). While adolescents and young adults are capable of and do engage in future orientation, their cognitions about the future tend more often to be positive rather than negative (Nurmi J-e 1992). This tendency is consistent with the notion that adolescents are motivated by reward rather than avoidance. It is not until later in life that individuals reflect on future states that they wish to avoid more frequently than desired future states (Nurmi J-e 1992). Researchers and clinicians may anticipate that a genetic finding of increased risk for a disease will have a greater impact if it is returned to an adolescent or young adult, since such a patient could change their behaviors for decades before the potential onset of a disease. Early efforts, therefore, will seem more likely to help patients avoid developing chronic conditions. However, the likelihood that adolescents and young adults would change their lifestyle to avoid a negative outcome that is decades away is small, given what is currently known about adolescent and young adult development.

## Conclusions

Current psychological theory related to motivation and subsequent behavior change does not support the expectation that disease risk information will be a useful tool for motivating patients to make behavior changes. Even if this information is available “for free” as an incidental finding from genotyping as a part of a research study, we can still not conclude that returning genetic information for this purpose is neutral. Even if a result can be provided with no additional laboratory confirmation, the time required to return the results is not “free.” Responsible providers will at a minimum need to provide framing information to help patients understand the meaning of the result, and to assure that excessively deterministic misconceptions are corrected. These efforts take valuable clinical time.

In the worst case, returning genetic risk information to patients could lead to a modest amount of distress and could lead to potentially inappropriate use of screening modalities (Bloss et al. 2011). The more common scenario, however, is simply that the provider and patient could have dedicated their time and attention to more effective preventive measures, such as motivational interviewing and focused goal-setting. The literature supporting such approaches is significantly more robust than the largely disappointing evidence on the use of genetic information to motivate behavior change (Hettema & Hendricks Dec 2010; Pollak et al. 2010; Resnicow et al. 2006).

Every use of clinical time involves a trade-off, and there is manifest interest in increasingly using this scarce resource to provide patients with genetic risk information. However, current empirical evidence provides more convincing support for non-genetic approaches to motivating patients. There is precious little evidence that would support replacing these approaches.

Given the scope of this article, we have not addressed the question of whether patients and research subjects have a right to access any and all genetic test results generated from their biological samples. The thrust of our argument would not necessarily preclude efforts to allow patients or research subjects to be given access to such results, such as through an online portal. However, even if patients have access to these genetic results, our analysis demonstrates that there will be little utility to spending clinical time to attempt to use this information to motivate behavior change in patients.

For the present, providers should continue to depend primarily on counseling approaches to motivating patients to make changes in their health behaviors. Over time, genetic risk information may be utilized to supplement these more traditional methods, but such a combination of approaches should be considered experimental. These efforts should take place within a clinical research context where their effects, positive and negative, can be evaluated carefully. Until the outcomes of such research can be used to inform practice, many genetic variants generated through research or clinical genetic testing should simply remain in wait. Efforts to realize the promise of Personalized Medicine should start with results that can more convincingly be regarded as actionable.
